# A novel RNA-mediated mechanism causing down-regulation of insulating promoter interactions in human embryonic stem cells

**DOI:** 10.1038/s41598-021-02373-1

**Published:** 2021-12-01

**Authors:** Yingjuan Liu, Simon G. Williams, Hayden R. Jones, Bernard D. Keavney, Mun-Kit Choy

**Affiliations:** 1 Division of Cardiovascular Sciences, The University of Manchester, Manchester, M13 9PT UK; 2 Manchester Heart Institute, Manchester University NHS Foundation Trust, Manchester, M13 9WL UK

**Keywords:** Biochemistry, Computational biology and bioinformatics, Genetics, Molecular biology, Stem cells

## Abstract

The genome-wide promoter interactome is primarily maintained and regulated by architectural proteins such as CTCF and cohesin. However, some studies suggest a role for non-coding RNAs (ncRNAs) in this process. We aimed to characterise the regulatory role of RNA-mediated promoter interactions in the control of gene expression. We integrated genome-wide datasets of RNA-chromatin and promoter-genome interactions in human embryonic stem cells (hESCs) to identify putative RNA-mediated promoter interactions. We discovered that CTCF sites were enriched in RNA-PIRs (promoter interacting regions co-localising with RNA-chromatin interaction sites) and genes interacting with RNA-PIRs containing CTCF sites showed higher expression levels. One of the long noncoding RNAs (lncRNAs) expressed in hESCs, *Syntaxin 18-Antisense 1* (*STX18-AS1*), appeared to be involved in an insulating promoter interaction with the neighbouring gene, *MSX1*. By knocking down *STX18-AS1*, the *MSX1* promoter-PIR interaction was intensified and the target gene (*MSX1*) expression was down-regulated. Conversely, reduced *MSX1* promoter-PIR interactions, resulting from CRISPR-Cas9 deletion of the PIR, increased the expression of *MSX1*. We conclude that *STX18-AS1* RNA antagonised local CTCF-mediated insulating promoter interactions to augment gene expression. Such down-regulation of the insulating promoter interactions by this novel mechanism may explain the higher expression of genes interacting with RNA-PIRs linked to CTCF sites.

## Introduction

Gene regulation involves the formation of three dimensional (3D) chromosomal interactions to bring distal genomic regulatory elements to spatial proximity of gene promoters^1,2^. The occurrence of these long-range chromosomal interactions is not only mediated by protein factors that are key players in maintaining genome architecture such as CCCTC binding factor (CTCF), Yin Yang 1 (YY1) and the Mediator and cohesin complexes, but also the widespread non-coding RNAs (ncRNAs) transcribed in the genome^3^. For example, *Thymocyte Differentiation factor* (*ThymoD*) ncRNA activates cohesin-dependent looping to juxtapose the enhancer and promoter of *B cell lymphoma/leukaemia 11b* (*Bcl11b*) gene into a single loop domain for T cell fate specifying expression^4^. *Functional intergenic repeating RNA element* (*Firre*), a long intergenic noncoding RNA (lincRNA) localises across a 5 Mb domain around its transcription site and this domain interacts with several trans-chromosomal loci^5^. Deletion of the *Firre* locus leads to the loss of the trans-chromosomal interactions, suggesting a role for *Firre* lincRNA in 3D chromosomal organisation^5^.

Although the mechanism by which ncRNAs facilitate long-range chromosomal interactions is largely unknown, it has been hypothesised that this function is made possible by their flexible modular domains that bind proteins, DNAs and other RNAs and bring these regulatory components to close proximity^6^. Architectural proteins such as the Mediator complex, CTCF and YY1 have been shown to associate with RNAs^7–10^. Depletion of Mediator’s subunits or activating ncRNAs reduces the chromatin looping between the activating ncRNAs and their target genes^7^, whilst mutations at CTCF’s RNA interaction sites cause the loss of chromatin loops^10^. In the case of cohesin, the positioning of the complex requires active transcription in the mouse genome^11^ and displacement of it depends on the ‘read-through’ transcription (extending past the 3′ ends of genes) in the host cell during the influenza A infection to decompact the locus^12^. This mechanism is an example of how the transcription itself, but not the resulting RNAs, can affect 3D genome organisation^3^.

In this study, we aimed to characterise the regulatory role of RNA-mediated promoter interactions in the control of gene expression. Recently, genome-wide and targeted methods have been devised to map RNA–DNA contacts in the genome, providing the experimental capability to understand the role of RNAs in chromosomal interactions^13–17^. Promoter-genome and RNA-genome mapping have been performed on human embryonic stem cells (hESCs) and their differentiated cells to understand the maintenance of pluripotency and the differentiation process^15,18,19^. Here we integrate promoter capture HiC (PCHiC) and mapping RNA-genome interactions (MARGI) datasets of H9 hESCs to identify and investigate putative RNA-mediated promoter interactions ^15,19^, and elucidate the role of a functionally important long noncoding RNA (lncRNA), *Syntaxin 18-Antisense 1* (*STX18-AS1*), in one of the promoter interactions of hESCs. SNPs in the region of the *STX18-AS1* gene have been associated with atrial septal defects of the heart in multiple studies^20–23^.

## Results

### CTCF and YY1 binding sites are enriched in RNA-PIRs

**Figure 1:**
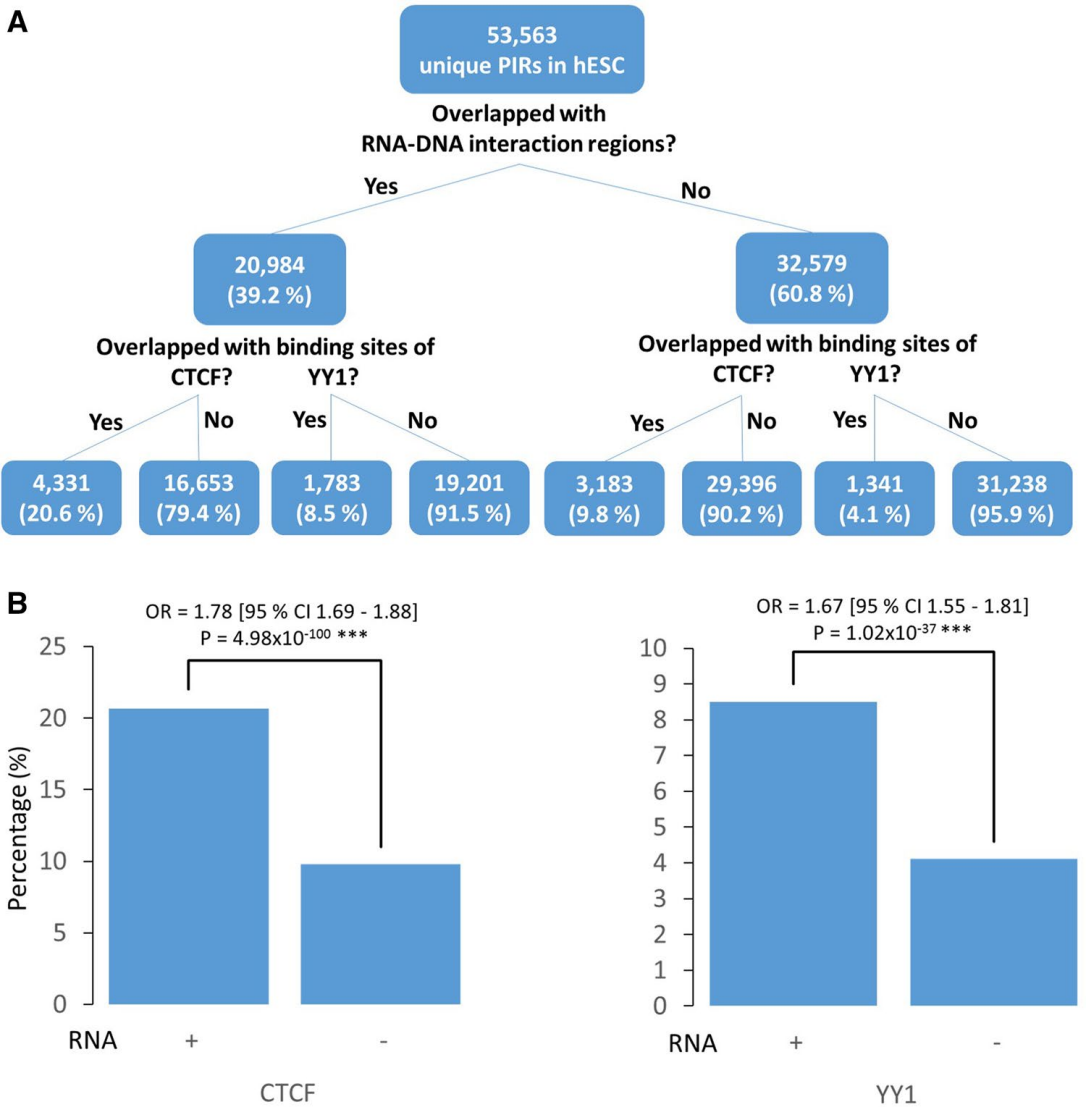
Enrichment of CTCF or YY1 binding sites in promoter interacting regions interacting with RNAs (RNA-PIRs). **(A)** Numbers and percentages of PIRs in human embryonic stem cells (hESCs) interacting with RNAs and containing binding sites of CTCF or YY1. **(B)** Odds Ratios (ORs) and p-values of CTCF or YY1 site enrichment were estimated from binary logistic regression analyses including PIR size as a covariate. ***p-values obtained from binary logistic regression analyses were lower than the p-values obtained from the same analyses performed with 1000 randomised CTCF or YY1 site sets (permutation P < 1.0x10^-3^).

We found that of the 53,563 unique promoter interaction regions (PIRs) in the genome of H9 hESCs previously reported^19^, 20,984 of them overlapped with genomic regions that are interacting with RNAs as identified through proximity MARGI (pxMARGI)^15^ (Fig. 1A). We hypothesised that RNAs play a role in the promoter-genome interactions, therefore we first investigated whether PIRs interacting with RNAs (RNA-PIRs) have enrichments for architectural protein binding such as CTCF and YY1. While CTCF is responsible for large chromosomal interactions to provide a 3D architecture for genes and their regulatory elements, more specific promoter-enhancer interactions are mediated by YY1^24^.

We obtained CTCF and YY1 binding sites of H1 hESCs from the Encyclopaedia of DNA Elements (ENCODE)^25,26^ and intersected them with the PIRs (Supplementary Table 1). 20.6% of RNA-PIRs contained CTCF binding sites, whereas only 9.8% of PIRs that are not interacting with RNAs (nonRNA-PIRs) had CTCF binding sites (odds ratio [OR] = 1.78; 95% CI = 1.69–1.88; P = 4.98 × 10^−100^). Similarly, 8.5% of RNA-PIRs contained YY1 binding sites, while 4.1% of nonRNA-PIRs contained these sites (OR = 1.67; 95% CI = 1.55–1.81; P = 1.02 × 10^–37^) (Fig. 1). The PIR size, which correlates with the probability of overlap, was included as a covariate in the binary logistic regression (Fig. 1B). We generated null distributions for the p-values of the binary logistic regression analyses by subjecting 1000 randomly generated genomic interval sets matching those in the CTCF or YY1 binding site sets to the same analyses. None of the p-values produced using randomised sets was lower than the actual p-values, hence the p-values of the binary logistic regression analyses were significantly lower than their null distributions (permutation P < 1.0 × 10^–3^).

PIRs are HindIII fragments and not the biological demarcations for the chromatin interacting regions. Therefore the intersection of PIRs and CTCF sites only indicates their physical proximity for the convenience of bioinformatic investigations, but not the actual sites where specific CTCF-mediated promoter interactions occur.

### Genes interacting with RNA-PIRs containing CTCF or YY1 binding sites have higher expression levels than those interacting with nonRNA-PIRs containing CTCF or YY1 binding sites

**Figure 2:**
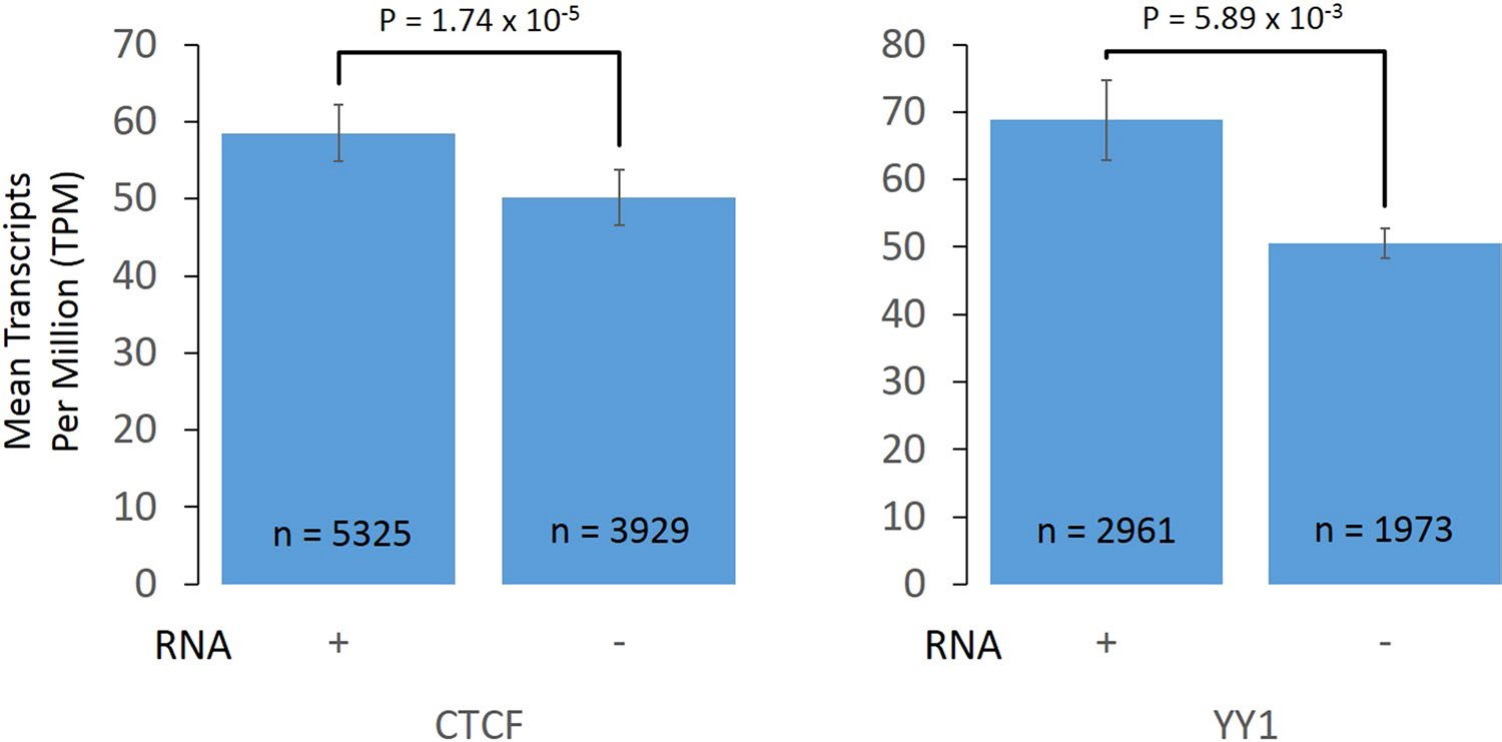
Average expression levels (mean Transcript Per Million [TPM]) of genes interacting with two categories of promoter interacting regions (PIRs): RNA-PIRs with CTCF or YY1 binding sites, and nonRNA-PIRs with CTCF or YY1 binding sites. Pairwise p-values were obtained from Mann–Whitney tests.

To understand if the expression of genes interacting with RNA-PIRs containing CTCF or YY1 sites are regulated differentially compared to those interacting with nonRNA-PIRs containing CTCF or YY1 sites, we obtained previously reported transcriptomic data of H9 hESCs^19^ and calculated average expression levels of genes interacting with the two categories of PIRs: (1) RNA-PIRs with CTCF or YY1 binding sites, or (2) nonRNA-PIRs with CTCF or YY1 binding sites (Fig. 2).

Between the categories, genes interacting with RNA-PIRs containing CTCF or YY1 sites appeared to have significantly higher expression levels (P = 1.74 × 10^–5^ for CTCF sites and P = 5.89 × 10^–3^ for YY1 sites), while genes interacting with nonRNA-PIRs with CTCF or YY1 sites had lower expression levels (Fig. 2). From these results, RNAs seemed to have up-regulated genes in the presence of CTCF or YY1, through long-range promoter-genome interactions in the nucleus.

### The co-regulation of RNAs and CTCF at PIRs plays an important role in biological processes in hESCs

**Figure 3:**
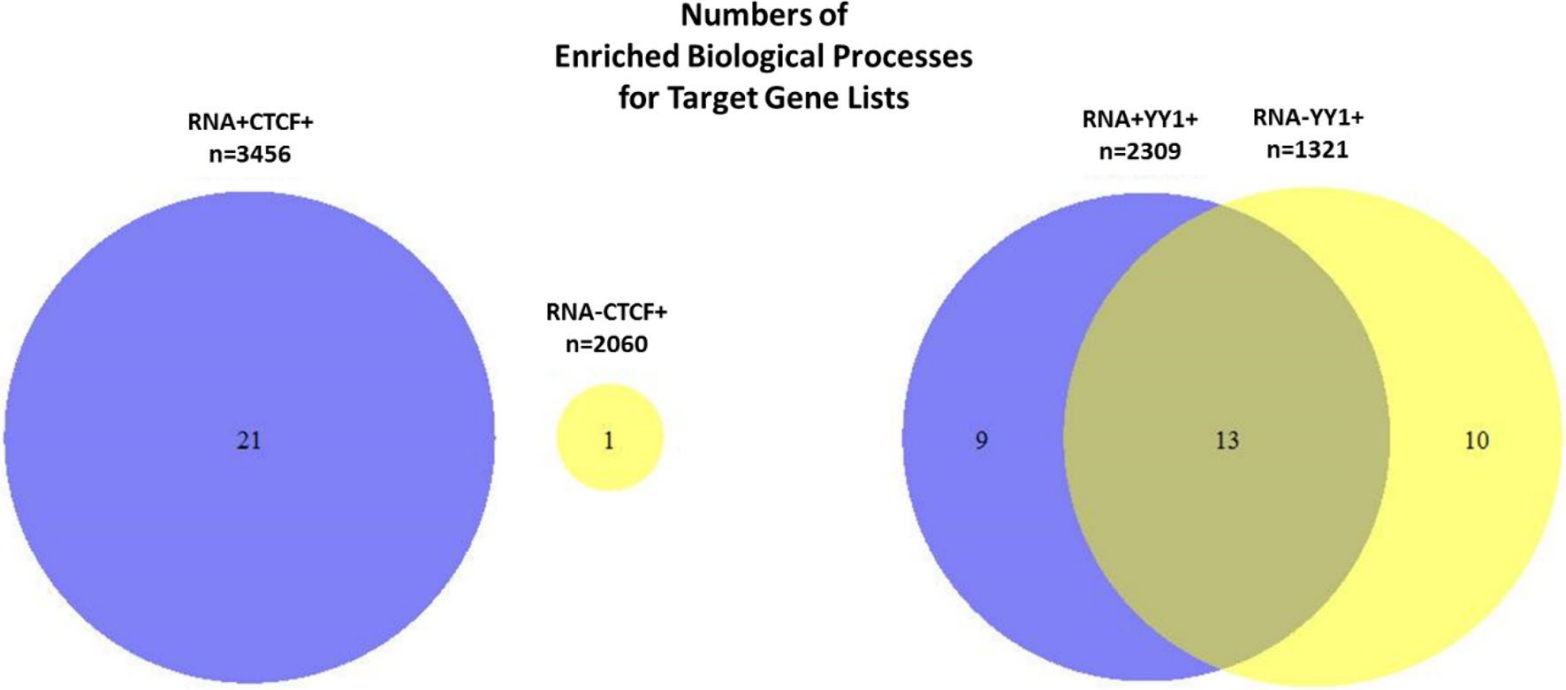
Numbers of biological processes (g:Profiler) that were significantly overrepresented (adjusted p < 0.01) in unique lists of genes interacting with RNA-PIRs or nonRNA-PIRs containing CTCF or YY1 sites.

Given that RNA-PIRs were enriched for more CTCF or YY1 sites and genes interacting with RNA-PIRs containing CTCF or YY1 sites exhibited higher expression levels, we hypothesised that the genes interacting with a PIR where both RNAs and CTCF/YY1 co-localised are the genes responsible for the biological functions of hESCs. We looked into the ontology of genes interacting with the RNA-PIRs containing CTCF or YY1 sites using g:Profiler^27^ (Supplementary Table 2). We compared the unique list of genes interacting with RNA-PIRs containing CTCF (3456 genes) or YY1 (2309 genes) sites with the unique list of genes interacting with nonRNA-PIRs containing CTCF (2060 genes) or YY1 (1321 genes) sites (Fig. 3).

g:Profiler returned the biological processes of genes that were significantly overrepresented in the unique gene lists (adjusted P < 0.01). The genes interacting with RNA-PIRs containing CTCF sites were significantly enriched in a wide range of development and cellular regulation processes (Supplementary Table 2). However, genes interacting with nonRNA-PIRs containing CTCF sites showed much fewer significant biological processes and no overlap when compared to genes interacting with RNA-PIRs containing CTCF sites, as only an “animal organ development” process was returned (Fig. 3). This suggests that the genes interacting with RNA-PIRs were involved in a wider range of the biological functions happening in hESCs than the genes interacting with nonRNA-PIRs.

Conversely, genes interacting with RNA-PIRs containing YY1 sites and genes interacting with nonRNA-PIRs containing YY1 sites belonged to similar biological processes. The two lists of genes shared 13 processes in development and cellular regulation (Fig. 3). Therefore, the results suggest that RNAs play a more important role in their co-regulation with CTCF than YY1. RNAs and CTCF, but not CTCF alone, regulate more genes involved in a broader range of biological functions in hESCs, whereas YY1, with or without RNAs, regulates genes belonging to a similar range of ontologies.

### *STX18-AS1* RNA represses an insulating promoter-PIR interaction of the neighbouring *MSX1* gene

**Figure 4:**
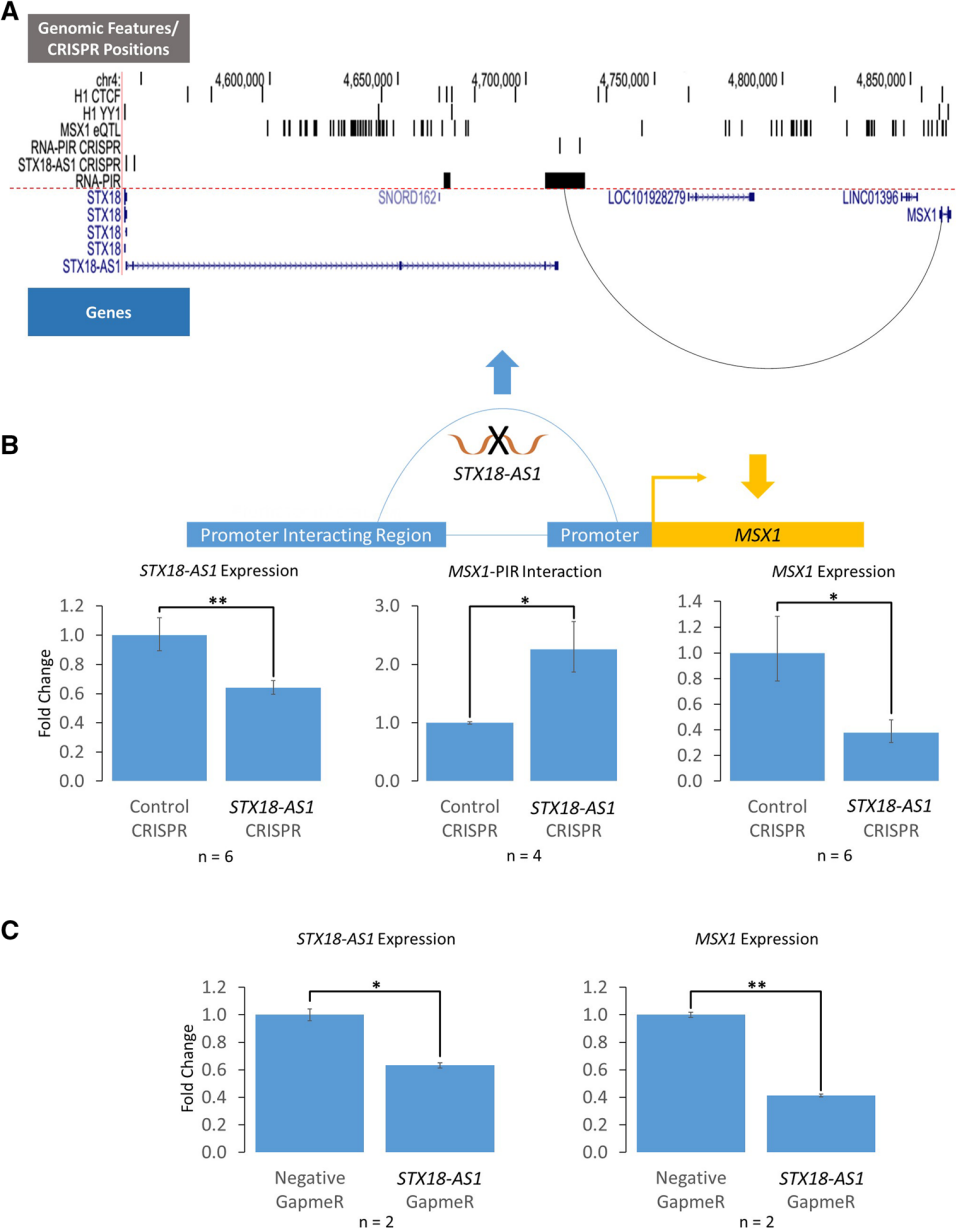

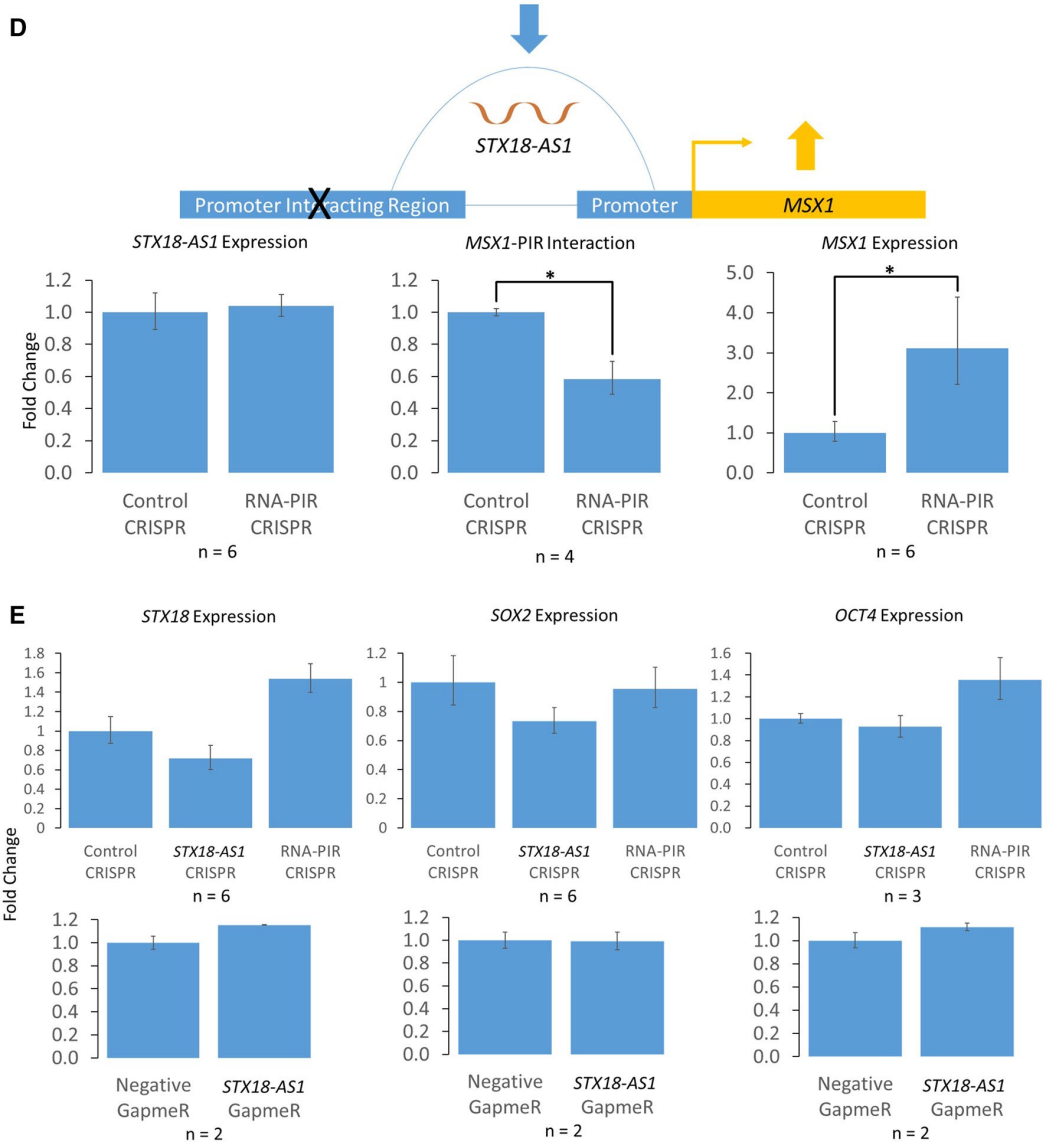
Chromosomal interaction between *MSX1* promoter and its promoter interacting region (PIR) mediated by a noncoding RNA, *STX18-AS1*. **(A)** Genomic locations of *MSX1* and *STX18-AS1* genes, CTCF and YY1 binding sites in H1 human embryonic stem cells (hESCs), GTEx expression quantitative trait loci (eQTLs) targeting *MSX1* gene, guide RNA (gRNA) sequences for deleting the first two exons of *STX18-AS1* gene and part of *MSX1* PIR using CRISPR-Cas9, and PIRs associated with RNAs (the *MSX1* promoter-PIR interaction was represented by an arc line). **(B)** Effects of *STX18-AS1* down-regulation by deleting part of *STX18-AS1* gene using CRISPR-Cas9 on *MSX1* expression and *MSX1* promoter-PIR interaction. **(C)** Effects of *STX18-AS1* down-regulation by a specific GapmeR on *STX18-AS1* and *MSX1* expression levels. **(D)** Effects of downregulated *MSX1* promoter-PIR interaction by deleting part of *MSX1* RNA-PIR using CRISPR-Cas9 on expression levels of *STX18-AS1* and *MSX1*. E) Effects of *STX18-AS1* and *MSX1* RNA-PIR CRISPR-Cas9 deletions, and *STX18-AS1* GapmeR on expression levels of *STX18*, *SOX2* and *OCT4*.*P < 0.05; **P < 0.01. All p-values were obtained from T tests with Bonferroni correction if multiple comparisons apply.

We further investigated the RNA-CTCF co-regulation in promoter interactions by experimentally manipulating a promoter interaction between the promoter of *Msh Homeobox 1* (*MSX1*), a transcription factor gene that plays a role in vertebrate embryogenesis^28^, and an RNA-PIR that is interacting with *Syntaxin 18-Antisense 1* (*STX18-AS1*), an lncRNA in hESCs. *STX18-AS1* gene contains genetic variants associated with atrial septal defects of the heart in genome-wide association studies (GWAS)^20–23^. As an RNA-PIR, We hypothesised that *MSX1* promoter-PIR interaction is insulating the expression of *MSX1* due to the association of RNA-RIRs with CTCF/YY1 sites as seen in Fig. 1B. In Fig. 4A, it is apparent that hESC CTCF sites are abundant between the PIR of *MSX1* and the promoter of *MSX1*, but YY1 sites are very sparse in the region, suggesting that CTCF is the main factor that mediates this chromatin interaction. We also hypothesised that *STX18-AS1* RNA upregulates *MSX1* expression, as the RNA presence in CTCF-mediated promoter interactions led to higher expression levels of the target gene of promoter interactions as seen in Fig. 2.

Firstly, we knocked down the expression of *STX18-AS1* RNA using paired guide RNA (gRNA) CRISPR-Cas9 technology^29^ to delete the first and second exons of *STX18-AS1* gene (Fig. 4A,B). We then checked the effect of *STX18-AS1* knock-down on the *MSX1* gene by quantifying *MSX1* gene expression using qPCR and *MSX1* promoter-PIR interaction using 3C-qPCR. *MSX1* expression was down-regulated but *MSX1* promoter-PIR interaction was up-regulated when *STX18-AS1* RNA was knocked down (Fig. 4B). These findings support the hypotheses that the *MSX1* promoter-PIR interaction acts as a CTCF-mediated interaction with an insulating effect on *MSX1* expression. Our results would be consistent with *STX18-AS1* RNA antagonising this interaction to upregulate the expression of *MSX1*. We confirmed this result by knocking down *STX18-AS1* using Antisense Locked Nucleic Acid (LNA) GapmeR technology^30^ targeting the second exon of *STX18-AS1* gene. *MSX1* was similarly down-regulated as a consequence (Fig. 4C)—the confirmation with GapmeR demonstrating that no additional regulatory element in the region removed by CRISPR-Cas9 was influencing the result of the CRISPR-Cas9 experiment. Thus, *STX18-AS1* RNA acted as a positive regulator for *MSX1* expression regardless of the knock-down technology used.

In order to verify the insulating effect of *MSX1* promoter-PIR interaction, we down-regulated the interaction by deleting part of the PIR that does not overlap with the *STX18-AS1* gene using paired gRNA CRISPR-Cas9 (Fig. 4A,D). The PIR deletion did not affect the expression of *STX18-AS1* RNA but reduced the amount of *MSX1* promoter-PIR interaction (Fig. 4D). In this case, the expression of *MSX1* was up-regulated, providing additional evidence that the *MSX1* promoter-PIR interaction is indeed a CTCF-mediated interaction with an insulating effect on *MSX1* expression.

When designing the gRNA constructs for deleting *STX18-AS1* gene and the PIR interacting with *MSX1*, regions rich in *MSX1*-targeting expression quantitative trait loci (eQTLs) were avoided (Fig. 4A). Therefore the experimental effects on *MSX1* caused by the deletions were aptly explained by the manipulations of *STX18-AS1* expression and *MSX1* promoter-PIR interaction, while no known cis-regulatory elements of *MSX1* were disrupted. All the CRISPR-Cas9 and GapmeR manipulations in this study did not significantly affect the expression of the upstream *STX18* gene or pluripotent marker genes *SOX2* and *OCT4* (Fig. 4E).

## Discussion

In this study we identified putative genome-wide RNA-mediated promoter-genome interactions in hESCs by integrating PCHiC and MARGI datasets and dissecting the role of an exemplar RNA in such an interaction. We provide evidence that RNAs co-regulate promoter interactions with CTCF. Firstly, binding sites of CTCF were more enriched in RNA-PIRs than nonRNA-PIRs. Secondly, genes interacting with RNA-PIRs containing CTCF sites had higher expression levels in the cells. Thirdly, genes interacting with RNA-PIRs containing CTCF sites, but not nonRNA-PIRs with CTCF sites, were overrepresented in a broad range of biological processes such as development and cellular regulation. On the other hand, genes interacting with PIRs containing YY1 sites belonged to a similar range of biological processes, with or without RNAs. Taking all the findings together, they suggest that RNAs and CTCF work together in maintaining and regulating promoter interactions.

By knocking down the RNA (*STX18-AS1*) of one of the putative RNA-mediated promoter-genome interactions (*MSX1* promoter-PIR interaction), the promoter interaction was intensified and the expression of the target gene (*MSX1*) was down-regulated. This suggests that the promoter interaction is repressive or insulating. The majority of promoter-genome interactions are maintained by architectural proteins such as CTCF and cohesin; indeed most promoter-genome interactions are lost when CTCF and cohesin are degraded^31^. Given the abundance of CTCF sites in the genomic region and CTCF being the major insulating factor to mediate promoter interactions, they suggest that the RNA (*STX18-AS1*) represses a CTCF-mediated insulating interaction with the promoter of the target gene (*MSX1*). The expression level of *MSX1* was up-regulated by knocking down the *MSX1* promoter-PIR interaction through deleting part of the PIR, indicating that the promoter interaction is both necessary and sufficient to regulate *MSX1* expression. Given the high expression levels shown by the genes interacting with RNA-PIRs containing CTCF sites, it supports the conclusion that the CTCF-mediated insulating promoter interactions can be suppressed by RNAs, just like the *MSX1* promoter-PIR interaction that insulates the *MSX1* expression is being suppressed by *STX18-AS1* RNA in hESCs.

## Materials and methods

### hESC maintenance

H9 (WA09) hESC line was a publicly available cell line obtained from WiCell, cultured in monolayers and maintained according to WiCell Feeder-Independent Pluripotent Stem Cell Protocols (SOP-SH-002) using mTeSR1 medium (STEMCELL Technologies).

### CRISPR-Cas9 and lentiviral transduction

gRNA sequences (provided in Supplementary Table 3) used to delete *STX18-AS1* gene and *MSX1* PIR in H9 hESC were cloned into pLentiCRISPRv2 (Addgene; plasmid 52961) according to published paired gRNA design^29^ using human U6 promoter for both gRNA sequences. The cloned pLentiCRISPRv2 and second-generation packaging plasmids psPAX2 (Addgene; plasmid 12260) and pMD2.G (Addgene; plasmid 12259) were co-transfected into HEK293T cells (cultured in DMEM (Gibco) supplemented with 10% FBS and 1 × penicillin–streptomycin at 37  ˚C and 5% CO_2_) in the proportion of 4:3:2 using Lipofectamine 2000 (Invitrogen) for lentivirus production. Lentivirus-containing media were collected 48 h after transfection, centrifuged to remove cell debris and 0.45 µm filtered before transducing H9 hESC monolayers (40–60% confluency) in the presence of 33% v/v mTESR1 media (STEMCELL Technologies), 8 µg/ml polybrene (Millipore) and 10 µM ROCK inhibitor. 24 h post-transduction, second transduction was repeated with fresh lentivirus-containing media. 24 h after the second transduction, the transduced H9 hESC were selected in 0.8 µg/ml puromycin media for 48 h. The strength of puromycin was less than the standard for better hESC survival, therefore the 48-h puromycin selection step was repeated every time the cells were passaged. The deletions of *STX18-AS1* gene and *MSX1* PIR were confirmed by PCR and Sanger sequencing (primers and product sizes are provided in Supplementary Table 3; results are in Supplementary Figs. 1 and 2). All CRISPR-Cas9 experiments were controlled by cells transduced with an empty pLentiCRISPRv2 plasmid.

### Antisense LNA GapmeR experiment

The GapmeR targeting *STX18-AS1*/*LOC100507266* lncRNA (NR_037888.1) was designed using EXIQON LNA GapmeR On-Line Design Tool (http://www.exiqon.com/gapmers). Both *STX18-AS1* and negative GapmeRs (details provided in Supplementary Table 3) were supplied by Qiagen/Exiqon at HPLC purification grade. GapmeRs were transfected to cells using Lipofectamine RNAiMAX according to the manufacture’s instruction (Invitrogen). Briefly for each 12-well of cells at ~ 60% confluency (1 ml of medium), 10 µl Lipofectamine RNAiMAX and 50 pmol GapmeR were added to cells and incubated at 37 °C for 24 h. The same procedure was repeated after 24 h and cells were incubated for another 24 h before the collection**.**

### 3C library preparation

3C libraries were prepared according to a published protocol^32^. In summary, 20 million hESCs were fixed by 2% formaldehyde (Agar Scientific; R1026) for 10 min at room temperature, quenched by cold 0.125 M glycine (5 min at room temperature followed by 15 min on ice), flash-frozen in liquid nitrogen and stored at − 80 °C. The cross-linked cells were lysed and the chromatin digested with HindIII at 37 °C for overnight. Then in-nucleus ligation was carried out at 16 °C for 4–6 h before reverse-crosslinking was achieved by overnight treatment of proteinase K at 65 °C. The DNA was purified by RNaseA treatment for 60 min at 37 °C, sequential phenol and phenol–chloroform extractions, and ethanol-precipitation at − 20 °C for overnight. On the following day, two further phenol–chloroform extractions and a second overnight precipitation were performed to further purify the DNA. The promoter interaction junctions between two HindIII fragments were confirmed by PCR and Sanger sequencing (primers and band sizes are provided in Supplementary Table 3; results for *MSX1* and *ERCC Excision Repair 3* (*ERCC3*) interactions (positive/internal control) are in Supplementary Figs. 3 and 4).

### Polymerase chain reaction (PCR)

Genomic DNA (gDNA) was extracted from cells using PureLink Genomic DNA Mini Kit (Invitrogen) and total RNA using Trizol (Invitrogen). First-strand complementary DNA (cDNA) was synthesised from DNase (Promega) treated total RNA using M-MLV Reverse Transcriptase (Promega) and both random hexamer and oligo(dT) primers (Promega). PCR was performed using OneTaq 2 × Master Mix (New England BioLabs) and the primers provided in Supplementary Table 3. For quantitative PCR (qPCR), gene expression was quantified by TaqMan assays (Applied Biosystems; assay details are provided in Supplementary Table 3) using a ViiA7 qPCR system (Applied Biosystems). For each qPCR reaction, ~ 40 ng cDNA templates were used with TaqMan Gene Expression Master Mix (Applied Biosystems; stock concentration 2 ×) and the TaqMan assays (Applied Biosystems; stock concentration 20 ×). Promoter interactions in 3C libraries were quantified using SYBR Green PCR Master Mix (Applied Biosystems) and primers (information provided in Supplementary Table 3) in a ViiA7 qPCR system (Applied Biosystems). Three replicates were done for each qPCR reaction with the programme of 95 °C for 10 min, 40 × (95 °C for 15 s, 60 °C for 1 min). *Importin 8* (*IPO8*) was included as the internal control for expression quantification, while *ERCC3* interaction was used as the positive/internal/positive control for interaction quantification^33^.

### Data processing and statistical analyses

RNA-chromatin interactions were obtained from http://systemsbio.ucsd.edu/margi/ (pxMARGI)^15^. To compare to the promoter-PIR interactions, coordinates in hg38 were converted to hg19 using UCSC liftover function (https://genome.ucsc.edu/). RNA or DNA regions with ambiguous mapping between genome builds or that mapped to ‘unlocalized’ or ‘unplaced’ regions were removed from further analyses. This resulted in 734,970 unique RNA–DNA interactions and 601,055 unique RNA-interacting DNA regions. Unique PIRs from H9 hESCs^19^ excluding interchromosomal interacting regions (53,563) were then overlapped with these RNA–interacting DNA regions. A total of 20,984 of these PIRs were found to overlap RNA-interacting DNA regions, whereas the remaining 32,579 PIRs had no evidence of RNA interaction from these data.

Promoter-PIR interactions with evidence of accompanying RNA interaction, and those without RNA interaction, were assessed for overlap with CTCF and YY1 sites using the ENCODE CTCF and YY1 sites from H1 hESC cells (Consortium, 2012; Consortium et al., 2020). CTCF or YY1 sites overlapping PIRs (RNA or non-RNA) were counted using a consensus of all 5 ENCODE CTCF and 2 ENCODE YY1 sets (downloadable from https://genome.ucsc.edu/; “Uniform TFBS” and “ENCODE 3 TFBS” tracks). For greater confidence in the overlaps, PIR and CTCF/YY1 overlaps were only counted if all datasets agreed (i.e. all 5 in the case of CTCF overlap and 2 in the case of YY1 overlap). Any degree of intersection between the PIRs and these high-confidence feature sets was included. We performed binary logistic regression, using PIR length as a covariate, to determine statistical significance of the difference between PIRs with/without RNA and CTCF/YY1 intersection. For negative controls, we generated consensus genomic intervals from the 5 CTCF and 2 YY1 datasets respectively. These were randomly shuffled within the genome using Bedtools (v2.27.1; https://bedtools.readthedocs.io/en/latest/). 1000 randomly generated interval sets were then assessed for their intersection with PIRs through the same statistical analysis as the actual data.

Gene promoters interacting with PIRs either associated with RNAs, CTCF or YY1 sites were assessed for the expression levels of their respective genes in H9 hESCs. H9 hESC RNA-seq dataset was downloaded from GEO (gene expression omnibus) accession GSE86821 (Freire-Pritchett et al., 2017). Raw data from the two replicates were pooled and transcript expression values determined using Kallisto (Bray et al., 2016) and the GRCh38 rel79 transcriptome from Ensembl (https://www.ensembl.org/). The mean expression values in terms of Transcripts Per Million (TPM) for genes identified in each overlapping PIR set (“RNA and CTCF”, “CTCF-only”, “RNA-only” and “neither”) were calculated and compared.

g:Profiler (https://biit.cs.ut.ee/gprofiler/gost)^27^ was used to identify the overrepresentations of biological processes in gene lists. eQTLs were obtained from GTEx V8 (https://gtexportal.org/home/).

Data from qPCR were analysed using the 2^(−ΔΔCt) method as previously described^34^. According to this method, means and standard errors are calculated from ΔCT values.

Standard statistical methods mentioned were performed with relevant R packages or other publicly available tools. Sample scripts and steps of this study are available on https://github.com/keavneylab/PIR-RNA-CTCF.

## Supplementary Information

Supplementary Figures.

Supplementary Tables.
