# Supplementary material for: A novel RNA-mediated mechanism causing down-regulation of insulating promoter interactions in human embryonic stem cells
Source: Sci Rep. 2021 Dec 1;11:23233. doi: 10.1038/s41598-021-02373-1 (PMC8636647; doi:10.1038/s41598-021-02373-1)

**A novel RNA-mediated mechanism causing down-regulation of insulating promoter interactions in human embryonic stem cells**

Yingjuan Liu^1^, Simon G. Williams^1^, Hayden R. Jones^1^, Bernard D. Keavney^1,2^, Mun-Kit Choy^1^*

^1^Division of Cardiovascular Sciences, The University of Manchester, Manchester, M13 9PT, UK

^2^Manchester Heart Institute, Manchester University NHS Foundation Trust, Manchester, M13 9WL, UK

*Corresponding author: munkit.choy@manchester.ac.uk

**Supplementary Figure 1. CRISPR-Cas9 deletion in *STX18-AS1* gene. A) PCR products of the region with (*STX18-AS1* CRISPR) and without the deletion (Control CRISPR). B) Sanger sequencing (forward and reverse) to show the gRNA sites in the deleted PCR product (*STX18-AS1* CRISPR). C) Complete view of the gel picture shown in A).**

**Supplementary Figure 2. CRISPR-Cas9 deletion in *MSX1* PIR. A) PCR products of the region with (*MSX1* PIR CRISPR) and without the deletion (Control CRISPR; too large to be effectively amplified). B) Sanger sequencing (forward and reverse) to show the gRNA sites in the deleted PCR product (*MSX1* PIR CRISPR). C) Complete view of the gel picture shown in A).**

**Supplementary Figure 3. *MSX1* promoter-PIR interaction. A) The PCR product of the interaction junction between HindIII fragments of *MSX1* promoter and its PIR. B) Sanger sequencing (forward and reverse) to show the sequence of the interaction junction. C) Complete view of the gel picture shown in A).**

**Supplementary Figure 4. *ERCC3* interaction (positive/internal control for quantifying *MSX1* promoter-PIR interaction). A) The PCR product of the interaction junction between HindIII fragments of *ERCC3* interaction. B) Sanger sequencing (forward) to show the sequence of the interaction junction. C) Complete view of the gel picture shown in A).**

**Supplementary Information:**

**Supplementary Table 1. Complete lists of the four categories of promoter interacting regions (PIRs) in human embryonic stem cells (hESCs) (human genome hg19): RNA-PIRs with CTCF or YY1 binding sites, RNA-PIRs without CTCF or YY1 binding sites, nonRNA-PIRs with CTCF or YY1 binding sites, and nonRNA-PIRs without CTCF or YY1 binding sites.**

**Supplementary Table 2. Biological processes (g:Profiler) that were significantly overrepresented (adjusted P < 0.01) in unique lists of genes interacting with RNA-PIRs or nonRNA-PIRs containing CTCF or YY1 sites.**

**Supplementary Table 3. Details of guide RNA (gRNA) and GapmeR sequences, primers (with expected PCR product sizes), and probes used in this study.**


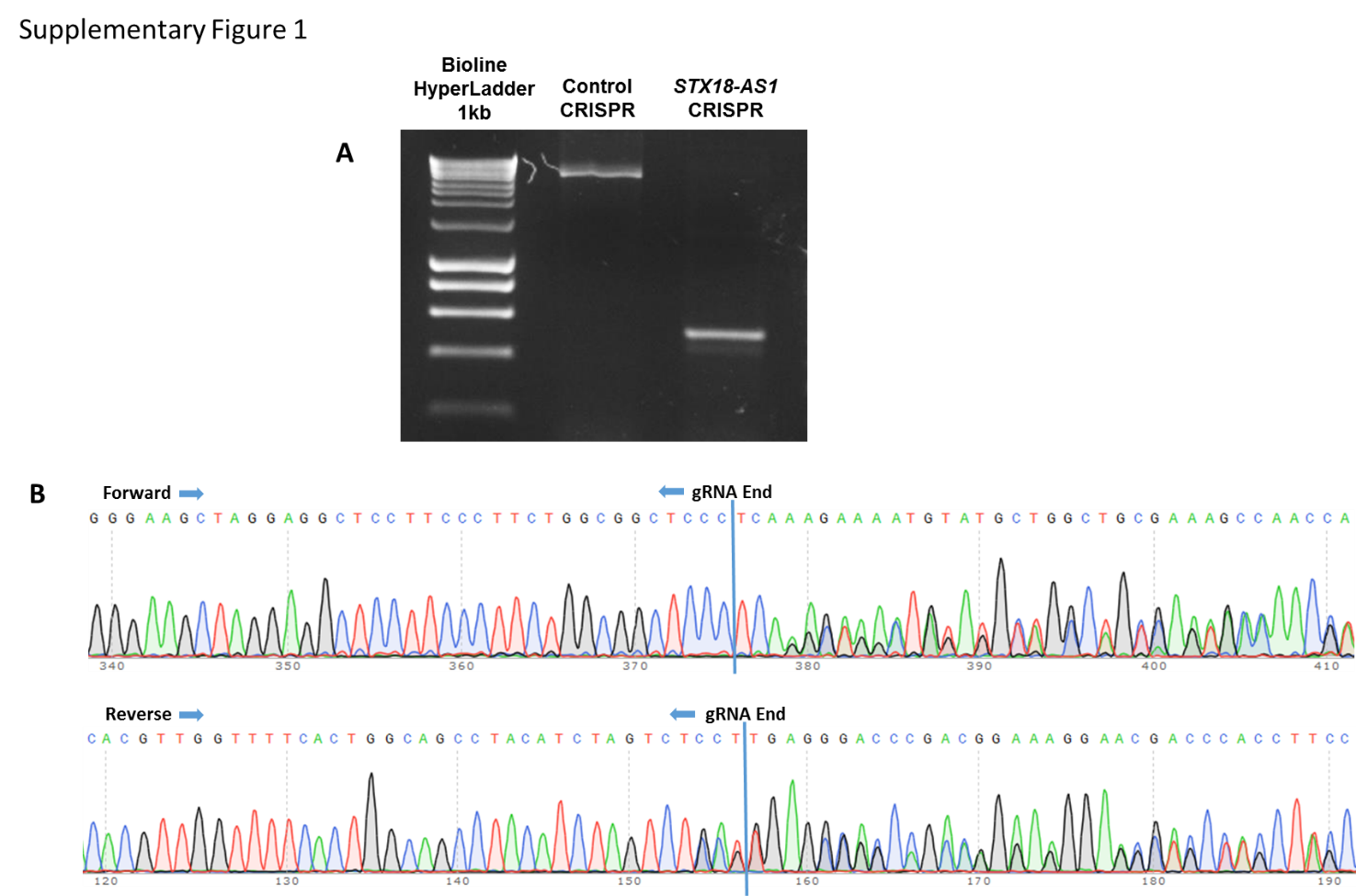


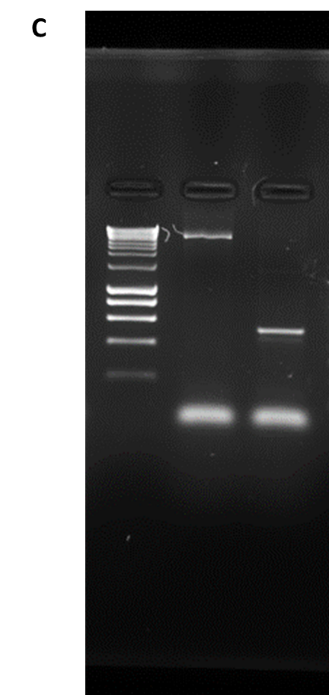


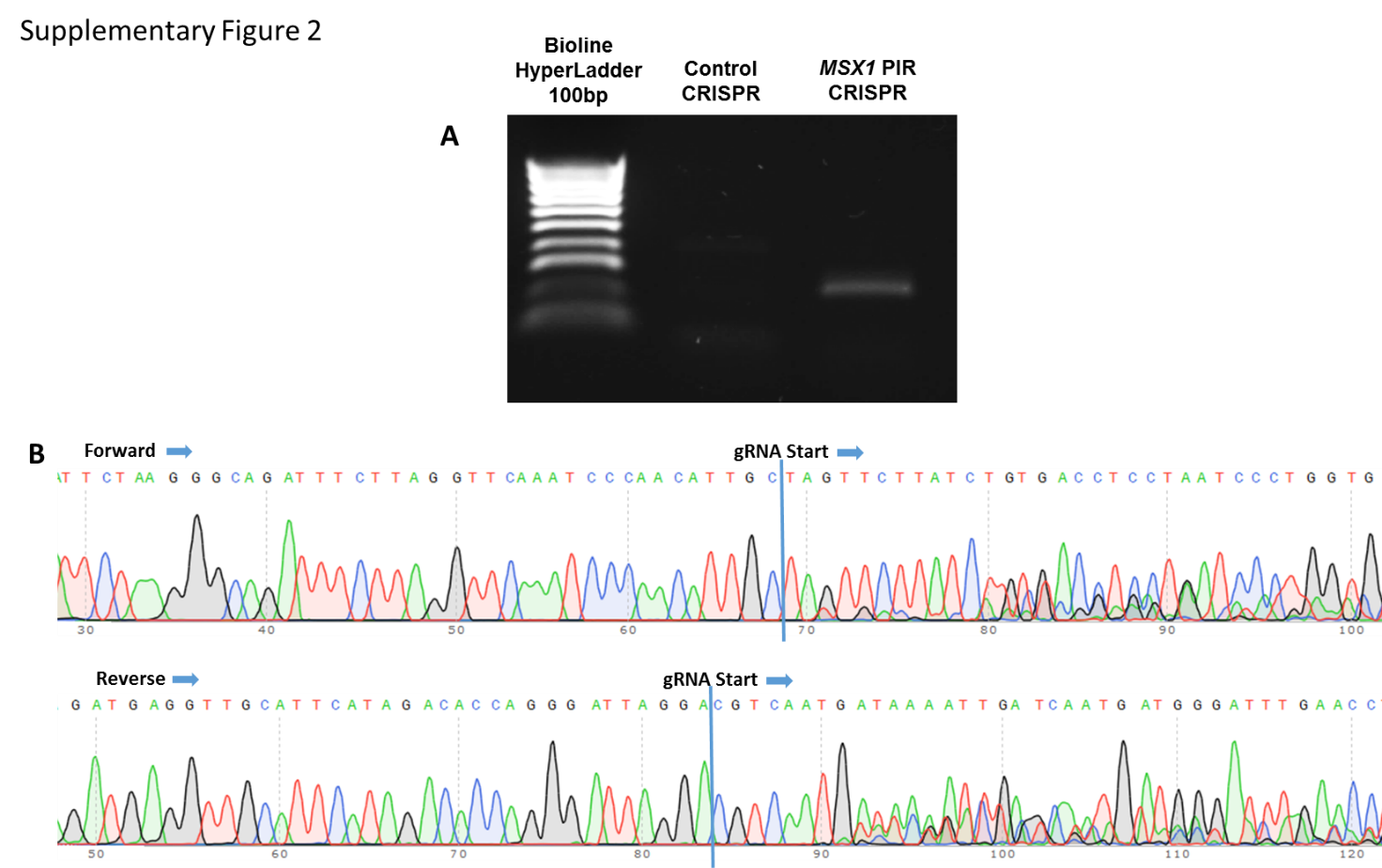


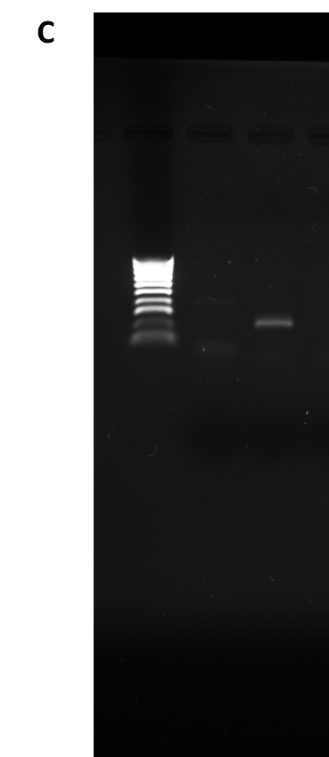


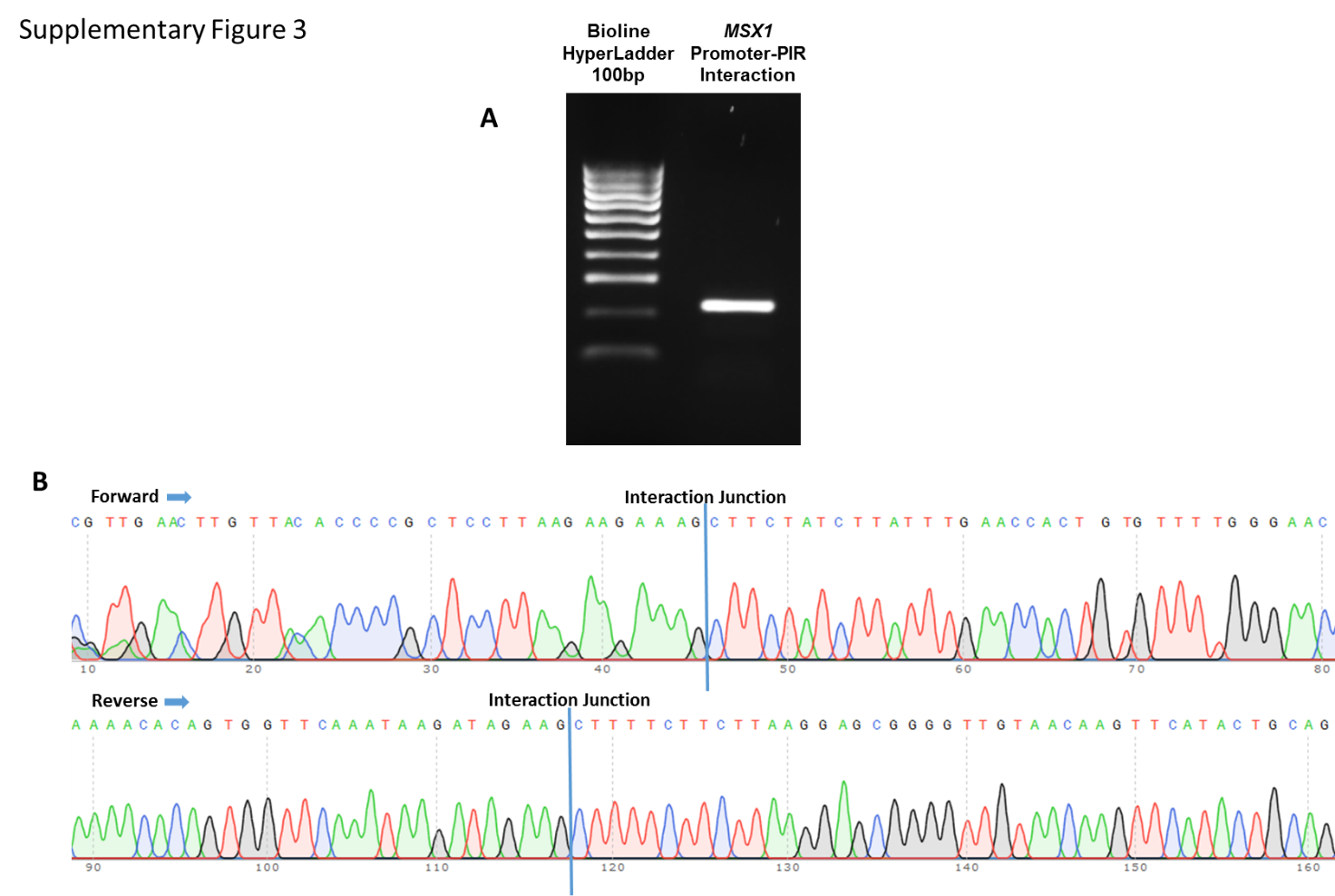


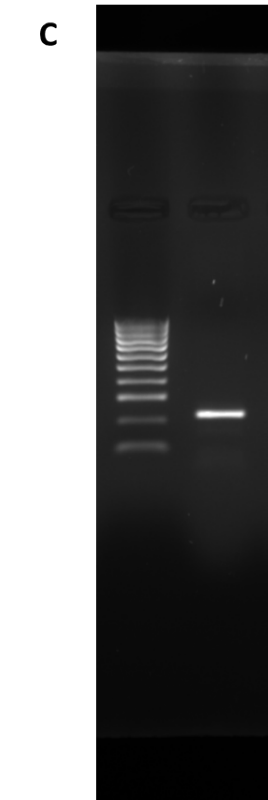


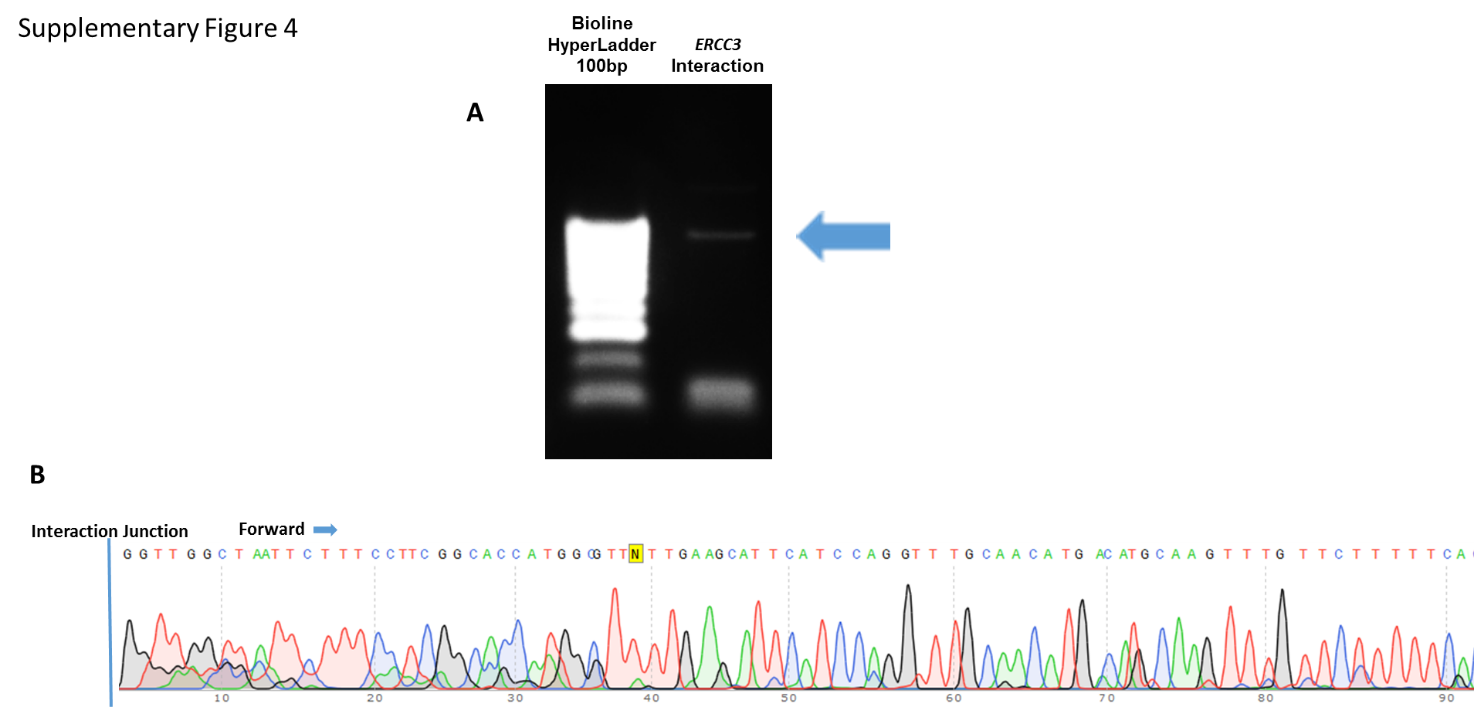


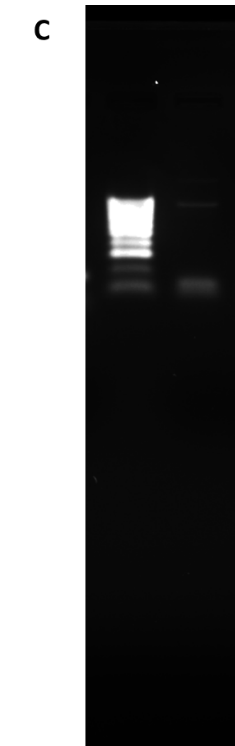

Supplement: Supplementary file 1 — Supplementary Figures. [file 41598_2021_2373_MOESM1_ESM.docx]
